# Characterization of papillary and clear cell renal cell carcinoma through imaging mass cytometry reveals distinct immunologic profiles

**DOI:** 10.3389/fimmu.2023.1182581

**Published:** 2023-08-11

**Authors:** Ameish Govindarajan, Nicholas J. Salgia, Haiqing Li, Daniela V. Castro, Tamara Mirzapoiazova, Brian Armstrong, Dan Zhao, Benjamin D. Mercier, Nazli Dizman, Neal Chawla, Zeynep Zengin, Luis Meza, Nishita Tripathi, Nicolas Sayegh, Alex Chehrazi-Raffle, Abhishek Tripathi, Sumanta K. Pal

**Affiliations:** ^1^ Department of Medical Oncology and Therapeutics Research, City of Hope Comprehensive Cancer Center, Duarte, CA, United States; ^2^ Jacobs School of Medicine and Biomedical Sciences, Buffalo, NY, United States; ^3^ Department of Immunology, Roswell Park Comprehensive Cancer Center, Buffalo, NY, United States; ^4^ Integrative Genome Core, Beckman Research Institute, City of Hope National Medical Center, Duarte, CA, United States; ^5^ Light Microscopy/Digital Imaging Core, Beckman Research Institute, City of Hope National Medical Center, Duarte, CA, United States; ^6^ Department of Gastrointestinal Medical Oncology, Division of Cancer Medicine, The University of Texas MD Anderson Cancer Center, Houston, TX, United States; ^7^ Department of Internal Medicine, Yale School of Medicine, New Haven, CT, United States; ^8^ Huntsman Cancer Institute-University of Utah Health Care, Salt Lake City, UT, United States

**Keywords:** cyTOF, IMC, clear cell renal cell carcinoma, papillary renal cell carcinoma, tumor microenvironment

## Abstract

**Objective:**

To characterize and further compare the immune cell populations of the tumor microenvironment (TME) in both clear cell and papillary renal cell carcinoma (RCC) using heavy metal-labeled antibodies in a multiplexed imaging approach (imaging mass cytometry).

**Materials and methods:**

Formalin-fixed paraffin-embedded (FFPE) baseline tumor tissues from metastatic patients with clear cell renal cell carcinoma (ccRCC) and papillary renal cell carcinoma (pRCC) were retrospectively requisitioned from an institutional biorepository. Pretreated FFPE samples from 33 RCC patients (10 ccRCC, 23 pRCC) were accessioned and stained for imaging mass cytometry (IMC) analysis. Clinical characteristics were curated from an institutional RCC database. FFPE samples were prepared and stained with heavy metal-conjugated antibodies for IMC. An 11-marker panel of tumor stromal and immune markers was used to assess and quantify cellular relationships in TME compartments. To validate our time-of-flight (CyTOF) analysis, we cross-validated findings with The Cancer Genome Atlas Program (TCGA) analysis and utilized the CIBERSORTx tool to examine the abundance of main immune cell types in pRCC and ccRCC patients.

**Results:**

Patients with ccRCC had a longer median overall survival than did those with pRCC (67.7 vs 26.8 mo, respectively). Significant differences were identified in the proportion of CD4^+^ T cells between disease subtypes (ccRCC 14.1%, pRCC 7.0%, p<0.01). Further, the pRCC cohort had significantly more PanCK^+^ tumor cells than did the ccRCC cohort (24.3% vs 9.5%, respectively, p<0.01). There were no significant differences in macrophage composition (CD68^+^) between cohorts. Our results demonstrated a significant correlation between the CyTOF and TCGA analyses, specifically validating that ccRCC patients exhibit higher levels of CD4^+^ T cells (ccRCC 17.60%, pRCC 15.7%, p<0.01) and CD8^+^ T cells (ccRCC 17.83%, pRCC 11.15%, p<0.01). The limitation of our CyTOF analysis was the large proportion of cells that were deemed non-characterizable.

**Conclusions:**

Our findings emphasize the need to investigate the TME in distinct RCC histological subtypes. We observed a more immune infiltrative phenotype in the TME of the ccRCC cohort than in the pRCC cohort, where a tumor-rich phenotype was noted. As practical predictive biomarkers remain elusive across all subtypes of RCC, further studies are warranted to analyze the biomarker potential of such TME classifications.

## Introduction

Cancers of the kidney and renal pelvis are anticipated to occur in 79,000 individuals in the United States in 2022, the majority of these cases constituting renal cell carcinoma (RCC) (1). RCC is a diverse disease comprising multiple biologically distinct histological features. The most frequent histological type (representing 70%-80% of cases) is clear cell RCC (ccRCC), which is driven by alterations in the Von Hippel-Lindau (*VHL*) gene (2). Somatic mutations in *VHL* occur in roughly half of patients with sporadic ccRCC and hypermethylation in an additional 10%-20% of cases, whereas germline alterations remain far less frequent. In its native form, *VHL* plays a role in the ubiquitination and subsequent degradation of hypoxia-inducible factor (HIF) (3). Dysregulated *VHL* therefore allows accumulation of HIF and transcription of downstream moieties such as vascular endothelial growth factor (VEGF), leading to tumor angiogenesis and proliferation.

Beyond ccRCC, the next most common histological type is papillary RCC (pRCC), a biologically distinct entity constituting 10%-15% of cases of RCC. The disease can be subdivided into type 1 and type 2 disease, and genomic studies have shown that both can carry alterations in the mesenchymal-epithelial transition (*MET*) protooncogene (although more frequent in type 1 disease) (4, 5). From a therapeutic perspective, pRCC remains a conundrum. Inherent to its biology, ccRCC responds well to VEGF-directed targeted therapies, of which multiple agents exist that are approved by the Food and Drug Administration (6–8). In addition, ccRCC shows sensitivity to immune checkpoint inhibitors (ICIs), including those blocking programmed death-1 (PD-1) and cytotoxic T-lymphocyte associated protein 4 (CTLA-4) (9). In contrast, pRCC shows markedly less sensitivity to VEGF-directed targeted therapies, although, interestingly, more specific MET inhibitors appear to show limited benefit in unselected patients (10, 11). Recently, randomized data have supported the role of dual VEGF and MET targeting in this disease, but response rates remain relatively low compared with those seen in ccRCC (12). Responses to ICIs in pRCC have been documented thus far only in single-arm studies, but in general, these rates are also lower than those observed in ccRCC (13).

Beyond the distinct canonical signaling pathways driving ccRCC and pRCC, it is possible that differences in the tumor immune microenvironment (TME) also drive the clinical behavior of these diseases. Various approaches have been undertaken to characterize this phenomenon, one of the first efforts being from Choueiri et al. (14), who reported variation in programmed death-ligand 1 (PD-L1) immunohistochemical expression across multiple non-clear cell histological subtypes. Although PD-L1 expression was minimally present on tumor cells, modest expression was seen on tumor immune cells in the microenvironment.

While the influence of PD-L1 expression in the TME is important, it’s critical to broaden the perspective on the factors that may impact the effectiveness of treatment strategies, especially concerning ICIs. Research into the field of immunotherapy has made it clear that the efficacy of ICIs in RCC is not solely dependent on the expression of PD-L1. A prime example is the CheckMate 214 trial, which revealed improved outcomes in RCC patients treated with a dual ICI regimen, irrespective of their PD-L1 status (9). This aligns with a meta-analysis of randomized clinical trials in metastatic RCC patients, where the predictive value of PD-L1 expression for ICI response was found to be uncertain (15). This observation underlines the multifaceted nature of tumor-host interactions within the TME, of which PD-L1 expression is but one aspect. It emphasizes the need for a broader understanding of the TME, including factors such as tumor mutational burden and immune infiltration, in order to refine treatment selection and improve patient outcomes. Together, these insights suggest a complex interplay of factors and highlight the importance of comprehensive study of the TME, beyond just canonical signaling pathways and PD-L1 expression. As such, more elaborate analyses of the TME have been pursued to uncover distinct immune profiles and their implications for treatment.

Recently, Synnott et al. (16) performed a broader immunohistochemical analysis encompassing multiple myeloid and lymphoid markers, their results suggest an increase in CD68^+^ macrophages in pRCC. Notably, neither study offered a direct juxtaposition of ccRCC and pRCC. In addition, methods now exist that have been used in an array of cancer types to more accurately characterize the TME (17–19). Building on this foundation, the main aim of our study was therefore to apply imaging mass cytometry (IMC) – a time-of-flight (CyTOF) tissue imaging technique that uses mass cytometry to characterize individual cells – in a cohort of patients with ccRCC and pRCC.

## Materials and methods

### Patient selection

Patients in the current study were retrospectively identified through an institutional database. Patients were eligible if they were 18 years and over with a pathologically confirmed diagnosis of either ccRCC or pRCC and clinical documentation of metastatic disease. Included in the study were those with available demographic details and treatment-related information, along with archival tissue sufficient for proposed correlative studies. Patients provided consent for use of tissue, as per institutional biospecimen repository processes. The protocol for tissue acquisition and use was reviewed and approved by an institutional review board.

### Molecular profiling of immune cells by using tissue samples

Formalin-fixed paraffin-embedded (FFPE) tumor tissue from primary or metastatic resection prior to therapy initiation was identified and sectioned. Our analysis primarily focused on analyzing tissues derived from the primary kidney site, encompassing the majority of patients within our cohort. The slides were simultaneously stained by multiple antibodies and analyzed by IMC technology with the Helios Hyperion Imaging System (Fluidigm Corporation, San Francisco, CA, USA) (20). Slides were dewaxed in xylene and hydrated in descending grades of ethanol (100%, 95%, 80%, 70%, 5 min each). The slides were then incubated in heated Tris/EDTA antigen retrieval solution, pH 9 (Dako, Agilent, Santa Clara, CA, USA), for 30 min and blocked with 3% bovine serum albumin (BSA) solution for 45 min at room temperature after being washed with deionized distilled water and Dulbecco’s phosphate-buffered saline (DPBS).

A custom panel of 11 metal-label antibodies (**Supplementary Table 1**) was generated in accordance with the protocol from Fluidigm. The slides were stained with the antibody cocktail in 0.5% BSA overnight at 4˚C in a hydration chamber. After being stained with antibodies, the slides were washed with 0.2% Triton-X in DPBS and then stained with Ir-Intercalator (1:600, Fluidigm) in DPBS for 30 min at room temperature. The stained slides were sent for imaging analysis after being rinsed and air dried. Tiled images were taken from the prepared slides on a Zeiss Observer Z1 with a 5´/0.16 NA objective and stitched by using Zeiss ZEN Blue software (Carl Zeiss Microimaging, White Plains, NY, USA). The images were oriented by using Image-Pro Premier 9.3.3 (Media Cybernetics, Baltimore, MD, USA) to locate and accurately select appropriate regions of interest of 500 μm × 500 μm for laser ablation and data acquisition with the Hyperion Imaging Cytometer (Fluidigm). The data for each marker were exported in TIFF format for downstream quantification.

**Figure 1:**
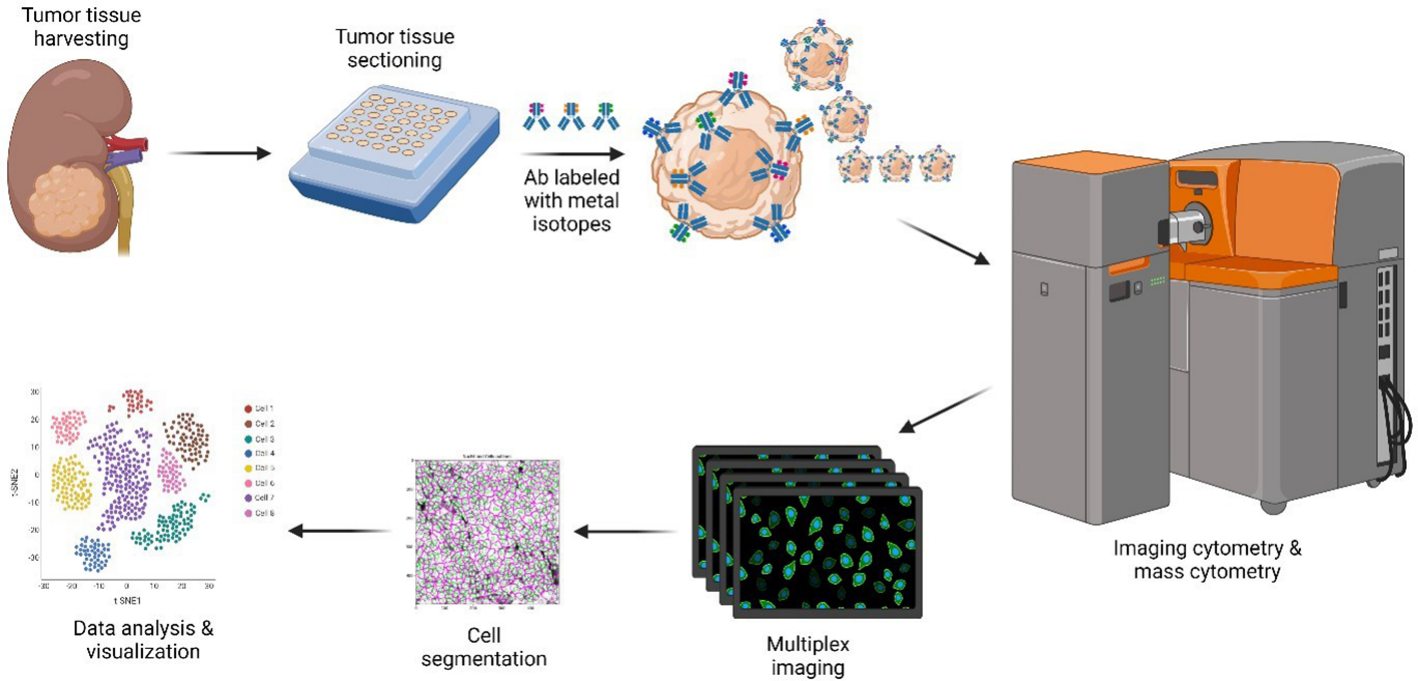
CyTOF methodology. Only the major steps are depicted. Resected ccRCC and pRCC samples stored in FFPE blocks were requisitioned, sectioned, and dewed. Heavy metal-antibody conjugates were used to stain tissue sections. Stained slides were visualized by using the Helios/Hyperion mass cytometer system for downstream data analysis and visualization. Created with BioRender.com. ccRCC, clear cell renal carcinoma; CyTOF, cytometry of time of flight; FFPE, formalin-fixed paraffin-embedded; pRCC, papillary renal cell carcinoma.

A combination of markers, including CD3, CD4, CD8a, FoxP3, CD68, Arginase-1, CD33, HLA-DR, Pan-Keratin (PanCK), PD-1, and PD-L1, was used to generate cell segmentation masks, which define the region of each individual cell and the background area on each image. Cell segmentation was performed with CellProfiler based on the mix of the marker images (21). Accurate cell counts and identification of spatial relationships, including co-localization and cell clustering, were analyzed with HistoCAT (22) and Partek Flow software (23). Single-cell measurements for all markers and cell spatial features were extracted from all images combined with the segmentation masks; single-cell level marker intensities of each sample were integrated by using a general linear model to remove sample variation. Multidimensional reduction was performed *via* t-distributed stochastic neighborhood embedding (t-SNE), allowing for visualization of multiplexed measurements within two-dimensional planes (**Figure 1**) (24). An unsupervised clustering algorithm, PhenoGraph, was used to classify the cell phenotypes from the abundances of all measured markers (25). From this analysis, five main cell types were identified: CD4 T cells (CD3 and CD4), CD8 T cells (CD3 and CD8), tumor cells (PanCK), macrophage cells (CD68), and “Others” (the latter being cells not identified with these specific markers). The cell population difference (counted as a percentage) of each cell type between pRCC and ccRCC patients was tested by using the R stat package Wilcoxon test (version 3.6.2), and the results were visualized by using the ggplot2 package (version 2.3) (26, 27).

### Immune cell abundance estimation using TCGA transcriptome data

We utilized the TCGA (The Cancer Genome Atlas Program) Biolinks package (version 2.28.3) in R to download the normalized RNA-seq expression data (FPKM) for primary tumors from TCGA KIRP (representing pRCC) and TCGA KIRC (representing ccRCC) (28). To simplify our computations, we filtered genes with low expression from the analysis.

The primary immune cell populations within these two cohorts were then assessed using CIBORSORTx, utilizing the default database of 22 immune cell types (29). The estimated abundance of CD4^+^ T cells was determined as the cumulative sum of CD4^+^ T naïve cells, CD4^+^ T memory activated cells, and CD4^+^ T memory resting cells. Additionally, we calculated the total monocyte cell count by combining monocytes, macrophages (M0, M1, M2), as well as both activated and resting dendritic cells.

### Renal cohort demographic and clinical factor analysis

Comparisons of overall survival between histological cohorts were performed by using Kaplan-Meier survival analyses with log-rank test. The hazard ratio was estimated by using the univariate Cox regression model. Comparisons of demographic and clinical factors between patient groups (pRCC vs ccRCC) were analyzed with Fisher’s exact test for categorical variables and the Wilcoxon test for continuous variables. Statistical analyses and data visualization were performed by using R’s finalfit package (version 1.0.4). All tests were two-sided, and p<0.05 was considered statistically significant (30).

## Results

### Patient characteristics

Specimens from 34 metastatic RCC patients were accessioned, stained, and analyzed *via* IMC. Among the 34 patients, 10 had ccRCC and 24 had pRCC. One patient specimen from the pRCC group was removed from cell population analysis due to cell segmentation failure caused by high background noise on the nuclear channels (191lr/193lr). Ultimately, 33 patients were included in the final analysis. The median age of the overall cohort at diagnosis was 62 (range 22-83), and the majority of patients (79%) were male. The median age at diagnosis in the ccRCC cohort was 59 (range 50-68) and in the pRCC cohort was 62 (range 22-83). The cohort’s IMDC (International Metastatic Renal Cell Carcinoma Database Consortium) status is determined according to Heng’s criteria (31). There were no notable differences observed between groups in terms of age, gender, IMDC risk status, or other clinicopathologic characteristics. Full demographic and clinicopathological characteristics of the cohort are summarized in **Table 1**.

**Table 1:** pRCC and ccRCC Cohort Overview.

Characteristic		ccRCC	pRCC	p-value
Age	Median (IQR)	59.0(54.2 to 65.0)	62.0(51.0 to 71.5)	0.44
Age Group	<=40	0 (0.0)	3 (13.0)	0.185
	>40 and <=65	8 (80.0)	10 (43.5)	
	>65	2 (20.0)	10 (43.5)	
Gender	Female	2 (20.0)	5 (21.7)	1
	Male	8 (80.0)	18 (78.3)	
Ethnicity	Hispanic/Latino	1 (10.0)	7 (30.4)	0.382
	Not Hispanic/Latino	9 (90.0)	16 (69.6)	
IMDC Risk Model	Favorable	1(10.0)	2(8.7)	0.825
	Intermediate/Poor	7(70.0)	18(78.3)	
	Unknown	2(20.0)	3(13.0)	
Pathology Collection Site	Kidney Primary	8(80.0)	17(73.9)	1
	Metastatic Site	2((20.0)	6(26.1)	
Line of Treatment	Median (IQR)	2.0 (1.0 to 3.5)	3.0 (1.0 to 3.0)	0.701
Immunotherapy	No	6 (60.0)	13 (56.5)	1
	Yes	4 (40.0)	10 (43.5)	
VEGF Inhibitor	No	2 (20.0)	10 (43.5)	0.259
	Yes	8 (80.0)	13 (56.5)	
MET Inhibitor	No	7 (70.0)	4 (17.4)	0.006
	Yes	3 (30.0)	19 (82.6)	
Drug Class	Chemotherapy	0 (0.0)	1 (4.3)	0.002
	Cytokine therapy	1 (10.0)	0 (0.0)	
	Immunotherapy	1 (10.0)	0 (0.0)	
	MET inhibitor	0 (0.0)	14 (60.9)	
	mTOR inhibitor	1 (10.0)	2 (8.7)	
	VEGF inhibitor	7 (70.0)	6 (26.1)	

ccRCC, clear cell renal carcinoma; IQR, interquartile range; MET, mesenchymal-epithelial transition; mTOR, mammalian target of rapamycin; pRCC, papillary renal cell carcinoma; VEGF, vascular endothelial growth factor; IMDC, International Metastatic renal cell carcinoma (RCC) Database Consortium.

**Figure 2:**
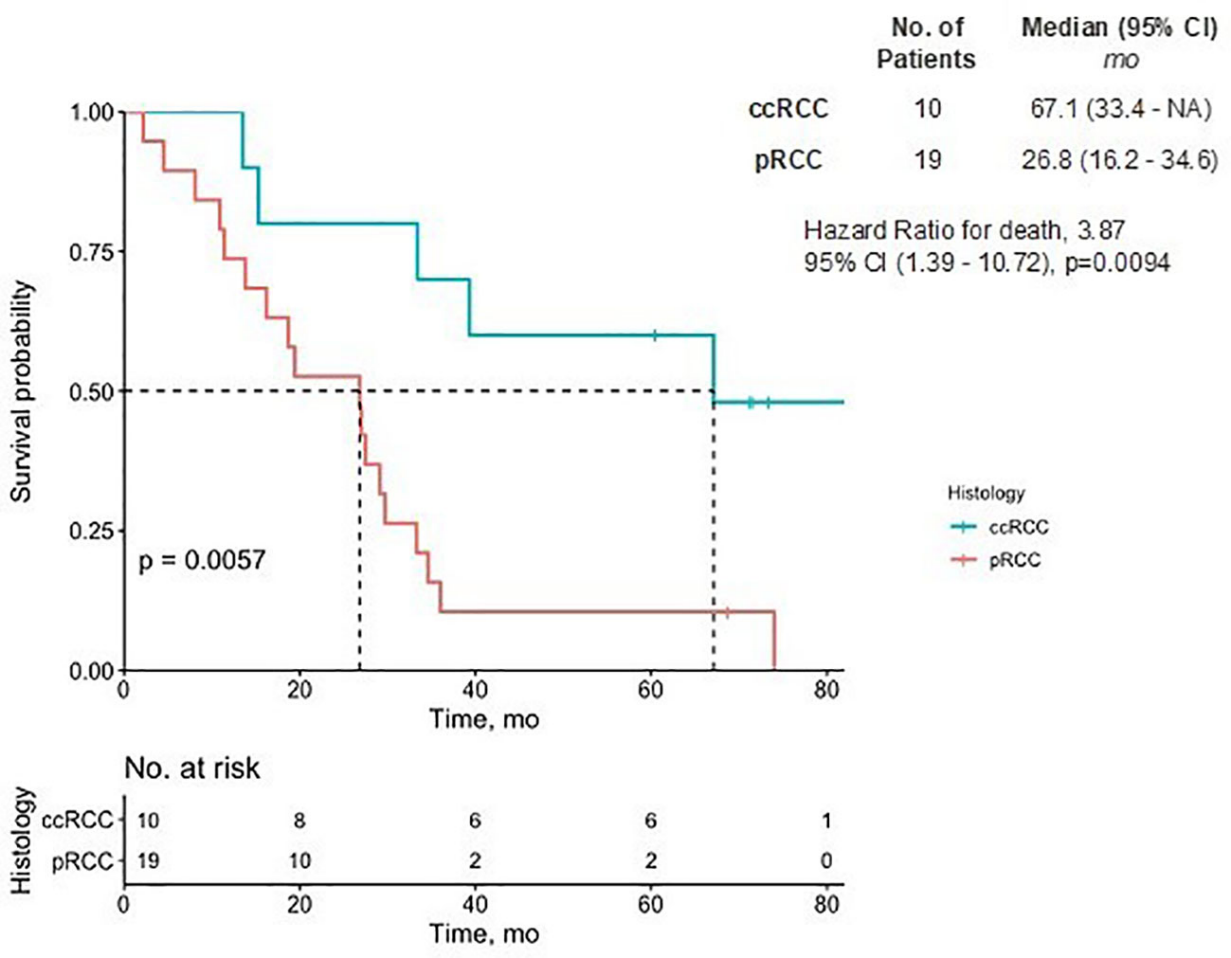
Overall survival of pRCC and ccRCC patients. The Kaplan-Meier estimate of overall survival up to 7 years is shown. *NA* denotes values that could not be estimated. There was a significant difference in the prognostic outcome between pRCC and ccRCC patients (p<0.01). CI, confidence interval; ccRCC, clear cell renal carcinoma; pRCC, papillary renal cell carcinoma.

In the ccRCC cohort, the mean number of prior lines of therapy was 2.5 (SD = 1.8), four patients having had prior ICIs, eight having received VEGF inhibitors, and three having received MET inhibitors. Among the pRCC cohort, the mean number of prior lines of therapy was 2.6 (SD=1.8), 10 patients having received ICIs, 13 having received VEGF inhibitors, and 19 having received MET inhibitors. There were no significant differences observed in the use of ICIs or VEGF inhibitor therapy between the two groups. The 7-year median overall survival was 67.1 months for the ccRCC patients and 26.8 months for the pRCC patients (**Figure 2**).

### CyTOF analysis

**Figure 3:**
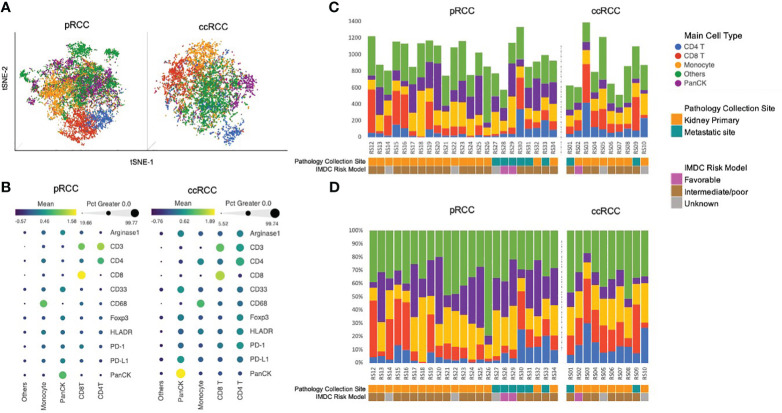
**(A)** Cell population annotation using CyTOF data. tSNE plot with cell annotation for 22,613 pRCC cells and 8,623 ccRCC cells based on their 11-marker intensity profile. **(B)** 11-marker intensity profile and cell annotation. The dot size represents the percentage of cells in this category that express the specific marker. The color of the dot represents the normalized marker intensity in a particular group of cells. Cells that did not have any specific markers were identified as *Others*. Those cells may not be represented by the 11 markers used in this study **(C)**. Cell population distribution by count for each patient. **(D)** Cell population distribution by composition for each patient. ccRCC, clear cell renal carcinoma; CyTOF, cytometry of time of flight; pRCC, papillary renal cell carcinoma; tSNE, t-distributed stochastic neighborhood embedding.

IMC data from 23 pRCC patients and 10 ccRCC patients were collected. A total of 22,613 cells were identified in the analysis from the pRCC cohort and 8,623 cells from the ccRCC cohort. To identify the specific cell type, we clustered pRCC and ccRCC CyTOF data separately on the basis of 11 markers. All pRCC cohort cells were arranged into 50 clusters in order to detect the difference in marker intensity among these cells. From the similarity of the mean intensity of the 11 markers, these 50 clusters of cells were merged and arranged into five groups (**Supplementary Figure 1A**), which included CD4^+^ T cells, CD8^+^ T cells, macrophage cells, PanCK^+^ tumor cells, and other cells. The ccRCC cohort cells were clustered into 17 clusters that were merged and characterized into the same five groups of cells (**Supplementary Figure 1B**) as in the pRCC cohort (see **Figure 3A**: tSNE plot with cell annotation for pRCC and ccRCC based on their normalized marker intensity profile, as noted in **Figure 3B**). Some cells (32% of pRCC cells, 35% of ccRCC cells) did not have any specific markers or marker profiles. As these cells may not represent the 11 markers used in this study, they were annotated as “Others.” The cell population distribution for each patient was collated by count and composition (**Figures 3C, D**).

### Cell population analysis of ccRCC vs pRCC

**Figure 4:**
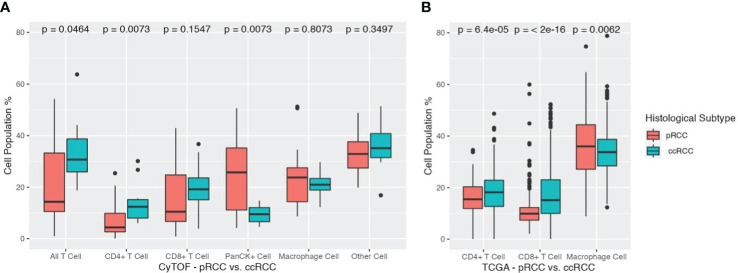
**(A)** Cell population differences across pRCC and ccRCC cohorts. Total T cells, CD4^+^ T cells, CD8^+^ T cells, PanCK^+^ cells, Macrophage cells, and Other cells are depicted for each cohort along with their corresponding p values. ccRCC, clear cell renal carcinoma; pRCC, papillary renal cell carcinoma. **(B)** Cell population differences across TCGA pRCC and ccRCC cohorts. Total T cells, CD4^+^ T cells, CD8^+^ T cells, monocyte/macrophage cells are depicted for each cohort along with their corresponding p values. ccRCC, clear cell renal carcinoma (TCGA KIRC); pRCC, papillary renal cell carcinoma (TCGA KIRP).

Analysis of differences in cell populations showed that ccRCC patients had significantly more CD3^+^ T cells than pRCC patients did (33.6% vs 21.8%, respectively, p<0.05). This result was largely driven by differences in the percentage of CD4^+^ T cells (ccRCC 14.1% vs pRCC 7.0%, p<0.01). Patients in the ccRCC cohort also had a greater, although nonsignificant, distribution of CD8^+^ T cells than the pRCC patients did (19.5% vs 14.8%, respectively, p=0.16). On the other hand, pRCC patients had significantly more PanCK^+^ tumor cells than the ccRCC patients did (24.3% vs 9.5%, respectively, p<0.01). There were no significant differences between the two groups in macrophage cell composition or in the other cells that were not represented by the 11 markers (**Figure 4A**).

To corroborate the findings from our CyTOF data analysis, we employed the CIBERSORTx tool to gauge the relative abundance of main immune cell types in both pRCC and ccRCC patients. The ensuing results underscored a remarkable alignment with our initial CyTOF findings. The TCGA cohort of patients with ccRCC exhibited a higher percentage of CD4^+^ T cells compared to those with pRCC, with respective percentages of 17.60% and 15.70% (p<0.01). This cohort also showed a greater prevalence of CD8^+^ T cells, with a percentage of 17.83% for ccRCC patients compared to 11.15% for pRCC patients (p<0.01). Interestingly, a converse trend was observed in the case of macrophages. The pRCC patients within the TCGA cohort presented a notably higher percentage of macrophage cells than their ccRCC counterparts, with proportions of 36.37% and 33.89%, respectively (p<0.01).

Given the extensive size of the TCGA cohorts, these differences in cell populations were all found to be statistically significant. The study’s cross-validation confirmed that ccRCC patients have elevated concentrations of both CD4^+^ and CD8^+^ T cells, contrasted with lower levels of monocyte/macrophage cells, as detailed in (**Figure 4B**).

## Discussion

Our study illustrates key differences in the TME between pRCC and with ccRCC, highlighting a distinction in lymphoid patterns, with an increase in T-cell distribution in ccRCC compared with that in pRCC. In addition, there were notable differences in PanCK, a marker of tumor cells, with a significantly increased population in pRCC compared with that in ccRCC. In our study, we bolstered our analysis by conducting cross-validation between our CyTOF analysis and the TCGA analysis. This further supported our findings, demonstrating that ccRCC patients exhibit elevated levels of CD4^+^ and CD8^+^ T cells, consistent with what we observed in the CyTOF analysis. To our knowledge, this is the first study to compare ccRCC and pRCC by using a mass cytometric approach.

Our study further supports the hypothesis that a high population of CD4^+^ and CD8^+^ T-cells within the TME in ccRCC may optimize the activity of cytotoxic T lymphocytes and potentially play an important role in this subtype’s sensitivity to ICIs (32). Previously, high T-cell populations were an indicator of poor prognoses in the setting of ccRCC; however, with the introduction of ICIs, robust responses are being established among patients with this subtype (33, 34). The observation that pRCC patients had a smaller percentage of T cells than did ccRCC patients may provide further insight into why pRCC shows marginal response rates to ICIs compared with those for ccRCC (13, 35).

Our results are in line with previous studies that highlighted an increased concentration of T cells within the TME through the use of CyTOF in the setting of ccRCC (36). Chevrier et al. (36) generated results showing that the main immune cell population in the TME were T cells, with a mean of 51% across samples from 73 ccRCC patients. This study included comprehensive TME analysis and stratified patients by determining the frequencies of the distinct immune cell phenotypes in concordance with clinical outcomes. In contrast, our study highlighted the individual markers for specific cell populations rather than the interrelation between immune cell populations within the TME. It would be prudent in future studies to consider this direction to better characterize the spatial architecture within the TME in concordance with clinical outcomes. Despite the use of CyTOF in ccRCC in the study by Chevrier et al. (36), the fact that they did not investigate other histological subtypes, such as pRCC, proved to the be a shortcoming.

In comparison to previous studies that focused on immunohistochemical staining, our novel application of CyTOF highlights a more refined approach to investigating the TME in non-ccRCC subtypes, which may yield potential biomarkers and help prognosticate clinical outcomes in the future (14, 37). This is in line with a recent study by Moldoveanu et al. (19), whereby CyTOF was used in the setting of melanoma and demonstrated the ability to characterize the granularity of the TME and identify correlates of ICI response.

Further, in the pRCC cohort, a greater number of PanCK^+^ tumor cells were found relative to those in the ccRCC cohort. A recent study investigated the immunostaining of PanCK^+^ tumor cells in 13,501 tumor samples from 121 different tumor types, including both pRCC and ccRCC histological subtypes (38). The results demonstrated an association between reduced PanCK^+^ tumor cells and advanced tumor staging, as well as higher metastatic risk. This contrasts with our findings of a higher level of PanCK^+^ tumor cells among the pRCC cohort, who subsequently had a poorer prognosis compared with that of the ccRCC cohort. Whereas these findings may be due to our smaller cohort size, they highlight the need for further investigation of PanCK^+^ cells and their potential to act as a predictive metastatic risk marker in RCC.

In our study, no significant differences were found in macrophage cell composition between the two histological subtypes. This finding is in contrast to that of Synnott et al. (16) who recently used immunohistochemical staining to characterize the TME in rare RCC histological types and found an increased expression of CD68^+^ macrophages in the tissue core and periphery of pRCC specimens (16). The role of macrophages in cancer progression is complex. In the presence of selected activating factors (e.g., lipopolysaccharides), macrophages can exert a tumoricidal effect (39, 40). However, multiple studies have suggested that macrophages can have an immunosuppressive effect in the tumor milieu, perhaps through the expression of PD-L1, PD-L2, and other factors that may quell the activity of adjacent CD8^+^ cells (40). Particularly relevant in the context of RCC, macrophages constitutively express HIF-1α and drive secretion of pro-angiogenic factors such as VEGF (41, 42). Taken together, these findings suggest that the increased presence of macrophages in pRCC could account for its decreased responsiveness to both ICIs and VEGF-targeted therapy. Given conflicting results regarding macrophages in the TME of pRCC, further research is needed to replicate findings and investigate possible clinical correlates with MET-directed therapies.

Perhaps the greatest limitation of our study was the large proportion of cells that were deemed non-characterizable. Although the nature of these cells remains unclear, with the CyTOF approach, we used a panel of 11 markers to differentiate cells, allowing for more thorough cellular characterization and providing a degree of confidence that these uncharacterized cells did not overlap with the immune cell populations of interest. Moreover, we reinforced the validity of our analysis by conducting additional cross-validation using data from the TCGA analysis. In future investigations, the utilization of multi-parametric spectral flow cytometry holds promise for enhancing the characterization of the TME, allowing for better understanding of its components. Furthermore, the incorporation of immunohistochemistry as an additional validation method could provide crucial spatial data that complements our CyTOF analysis and TCGA bulk RNA datasets. Given the heterogeneity of the tissue sample, our investigation was limited to analyzing a small tumor enriched region. However, for future studies, it would be advantageous to assess multiple regions per sample. Moreover, it is important to consider incorporating an evaluation of the surrounding normal population within the lymphoid tissues in addition to our primary focus on the tumor-enriched areas. This approach would provide valuable insights in forthcoming research endeavors. Finally, our study was limited by the sample size, although these high-dimensional results from over 20 distinct pRCC specimens nonetheless offer a novel perspective of this rare disease.

## Conclusions

This study offers novel insight into the TME of ccRCC and pRCC by using the CyTOF approach. Notably, important differences emerged in the TME between the two histological subtypes, potentially providing insight into their differential response to therapy and a foundation for further investigation that might identify prognostic biomarkers of clinical response. The TME remains a relatively unexplored domain, with a dynamic interplay between immune, endothelial, epithelial, and carcinoid compartments and providing robust opportunities for future research in RCC and its histological subtypes. Future clinical trials should integrate novel techniques that interrogate the TME and its interrelation between phenotypically distinct immune cell populations. This strategy may yield potential predictive biomarkers across distinct histological subtypes in RCC, guiding treatment decisions and providing impetus for novel drug discoveries.

## Data availability statement

The raw data supporting the conclusions of this article will be made available by the authors, without undue reservation.

## Ethics statement

The studies involving human participants were reviewed and approved by IRB# 19521 City of Hope Institutional Review Board. The ethics committee waived the requirement of written informed consent for participation.

## Author contributions

AG: Study concept and design, Acquisition of data, Analysis and interpretation of data, Statistical analysis, Drafting of the manuscript, Final approval of manuscript, and had full access to all the data in the study and take responsibility for the integrity of the data and the accuracy of the data analysis. NJS: Study concept and design, Acquisition of data, Analysis and interpretation of data, Statistical analysis, Drafting of the manuscript, Final approval of manuscript. HL: Study concept and design, Acquisition of data, Analysis and interpretation of data, Statistical analysis, Drafting of the manuscript, Final approval of manuscript. DC: Analysis and interpretation of data, Drafting of the manuscript, Final approval of manuscript. TM: Analysis and interpretation of data, Final approval of manuscript. BA: Analysis and interpretation of data, Final approval of manuscript. DZ: Analysis and interpretation of data, Final approval of manuscript. BM: Analysis and interpretation of data, Final approval of manuscript. ND: Analysis and interpretation of data, Final approval of manuscript. NC: Analysis and interpretation of data, Final approval of manuscript. ZZ: Analysis and interpretation of data, Final approval of manuscript. LM: Analysis and interpretation of data, Final approval of manuscript. NT: Analysis and interpretation of data, Final approval of manuscript. NS: Analysis and interpretation of data, Final approval of manuscript. AC-R: Analysis and interpretation of data, Final approval of manuscript. AT: Analysis and interpretation of data, Final approval of manuscript. SP: Study concept and design, Acquisition of data, Statistical analysis, Analysis and interpretation of data, Drafting of the manuscript, Provision of study material or patients, Final approval of manuscript, Supervision. All authors contributed to the article and approved the submitted version.
