# Supplementary figures and images for: Characterization of papillary and clear cell renal cell carcinoma through imaging mass cytometry reveals distinct immunologic profiles
Source: Front Immunol. 2023 Aug 11;14:1182581. doi: 10.3389/fimmu.2023.1182581 (PMC10457014; doi:10.3389/fimmu.2023.1182581)

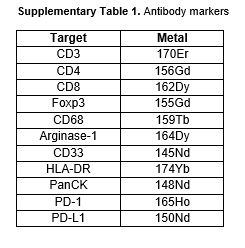

Supplement: Supplementary file 1 [file Image_1.jpg]
