# Supplementary material for: Characterization of papillary and clear cell renal cell carcinoma through imaging mass cytometry reveals distinct immunologic profiles
Source: Front Immunol. 2023 Aug 11;14:1182581. doi: 10.3389/fimmu.2023.1182581 (PMC10457014; doi:10.3389/fimmu.2023.1182581)
Supplement: Supplementary file 2 [file Table_1.docx]

**Appendix A**

**Supplementary Table 1.** Antibody markers

| **Target** | **Metal** |
| --- | --- |
| CD3 | 170Er |
| CD4 | 156Gd |
| CD8 | 162Dy |
| Foxp3 | 155Gd |
| CD68 | 159Tb |
| Arginase-1 | 164Dy |
| CD33 | 145Nd |
| HLA-DR | 174Yb |
| PanCK | 148Nd |
| PD-1 | 165Ho |
| PD-L1 | 150Nd |

**Supplementary Figure Legend:**

**Supplementary Fig. 1a and b.** pRCC and ccRCC cell clusters, marker intensity profile, and annotation. The 11-marker intensity profile and cell annotation for pRCC and ccRCC cells. The dot size represents the percentage of cells in this category expressing the specific marker. The color of the dot represents the marker intensity in that cluster of cells. ccRCC = clear cell renal carcinoma; pRCC = papillary renal cell carcinoma.
